# OMIC Technologies and Vaccine Development: From the Identification of Vulnerable Individuals to the Formulation of Invulnerable Vaccines

**DOI:** 10.1155/2019/8732191

**Published:** 2019-04-28

**Authors:** Nicola Cotugno, Alessandra Ruggiero, Veronica Santilli, Emma Concetta Manno, Salvatore Rocca, Sonia Zicari, Donato Amodio, Manuela Colucci, Paolo Rossi, Ofer Levy, Federico Martinon-Torres, Andrew J. Pollard, Paolo Palma

**Affiliations:** ^1^Academic Department of Pediatrics, Research Unit of Congenital and Perinatal Infection, Children's Hospital Bambino Gesù, Rome, Italy; ^2^Laboratory of Nephrology, Department of Rare Diseases, Children's Hospital Bambino Gesù, Rome, Italy; ^3^Precision Vaccines Program, Division of Infectious Diseases, Department of Medicine, Boston Children's Hospital, Boston, MA, USA; ^4^Broad Institute of MIT & Harvard, Cambridge, MA, USA; ^5^Translational Pediatrics and Infectious Diseases, Department of Pediatrics, Hospital Clínico Universitario de Santiago, Santiago de Compostela, Galicia, Spain; ^6^Grupo de Investigación en Genética, Vacunas, Infecciones y Pediatría (GENVIP), Instituto de Investigación Sanitaria de Santiago and Universidade de Santiago de Compostela (USC), Galicia, Spain; ^7^Oxford Vaccine Group, Department of Paediatrics, University of Oxford and the NIHR Oxford Biomedical Research Centre, Oxford, UK

## Abstract

Routine vaccination is among the most effective clinical interventions to prevent diseases as it is estimated to save over 3 million lives every year. However, the full potential of global immunization programs is not realised because population coverage is still suboptimal. This is also due to the inadequate immune response and paucity of informative correlates of protection upon immunization of vulnerable individuals such as newborns, preterm infants, pregnant women, and elderly individuals as well as those patients affected by chronic and immune compromising medical conditions. In addition, these groups are undervaccinated for a number of reasons, including lack of awareness of vaccine-preventable diseases and uncertainty or misconceptions about the safety and efficacy of vaccination by parents and healthcare providers. The presence of these nonresponders/undervaccinated individuals represents a major health and economic burden to society, which will become particularly difficult to address in settings with limited public resources. This review describes innovative and experimental approaches that can help identify specific genomic profiles defining nonresponder individuals for whom specific interventions might be needed. We will provide examples that show how such information can be useful to identify novel biomarkers of safety and immunogenicity for future vaccine trials. Finally, we will discuss how system biology “OMICs” data can be used to design bioinformatic tools to predict the vaccination outcome providing genetic and molecular “signatures” of protective immune response. This strategy may soon enable identification of signatures highly predictive of vaccine safety, immunogenicity, and efficacy/protection thereby informing personalized vaccine interventions in vulnerable populations.

## 1. Introduction

Vaccine-preventable disease (VPDs) pose an ongoing threat to health worldwide which can be avoided by protective and long-lasting vaccination coverage. Vaccines already prevent 3 million deaths every year by providing immunity against relevant pathogens. Nonetheless, current coverage rates are suboptimal especially in the so-called “vulnerable populations” (VPs) which include newborns, preterm infants, pregnant women, and elderly individuals as well as those patients affected by chronic and immune compromising medical conditions [1]. There are various reasons for this undervaccination, including lack of awareness of vaccine-preventable diseases and uncertainty or misconceptions about the safety and efficacy of vaccination among vulnerable patients, parents, and healthcare providers. Furthermore, in these VPs, the immune responses obtained with currently available vaccines and schedules can be inadequate leading to lower protection compared with healthy individuals [1, 2]. This situation represents a major health and economic burden to society, which will become particularly difficult to address in settings with limited public resources. As a consequence, renewed attention and innovative strategies are required to overcome the many challenges faced by public health authorities to improving the efficacy of immunization programs [3]. Two strategies are needed: (1) improve current vaccination approaches by addressing education and management of vaccine hesitancy and (2) develop innovative tools that enable explanation of mechanisms behind low or no responsiveness to current vaccine regimens in these groups and design specific interventions accordingly (i.e., booster doses of vaccines and/or tailoring adjuvantation systems for vaccine formulations targeted to specific subpopulations). In this review, we will mainly focus on innovative genomic and transcriptomic tools that can identify specific host characteristics defining nonresponder individuals for whom specific interventions might be needed.

### 1.1. Low Vaccination Coverage in Vulnerable Populations: Some Concerning Data

Low vaccination coverage in vulnerable groups increases the risk of developing vaccine-preventable diseases with higher morbidity and mortality [1]. The fact that vaccination rates among at-risk populations remain low despite national and international recommendations indicates a continuing failure to provide appropriate standards of care. One example is represented by maternal immunization against influenza, pertussis, and tetanus, which has the untapped potential of protecting the infant, which remains low in European pregnant women (38-50%) [4]. As a consequence, pertussis cases and outbreaks have increased over the last few decades with ~1400 cases of whooping cough documented in children < 6 months of age in the US that lead to hospitalization in 44.3% of cases in 2016 CDC [5]. Additionally, infants < 6 months who experience influenza virus infection have the highest rates of hospitalization and death of all children especially if born preterm [6]. Indeed, as current influenza vaccines are licensed for use in those from 6 months of age, those less than 6 months of age are too young to receive routine influenza vaccination with protection relying on that conferred by a vaccinated mother. Another example of low vaccination coverage is represented by elderly populations: in developing countries, the need for better vaccination coverage of aging populations is well recognised (reviewed in [1]). In the US, coverage among people aged ≥65 years was 67% for the influenza vaccine in the 2014–2015 and 55–60% for tetanus and pneumococcal vaccines in 2013, while the coverage rate for herpes zoster vaccination among those aged ≥60 years was only 24%. In most other countries, rates are far lower (reviewed in [1]). Furthermore, patients who are immunocompromised are also undervaccinated [1, 7]. This diverse group of patients includes patients with primary immunodeficiency, human immunodeficiency virus (HIV) infection, transplantation, cancer, asplenia, and autoimmune inflammatory diseases treated with immunosuppressive medications (corticosteroid therapy, immunomodulatory medications, or biological agents) [8–11].

Vaccine hesitancy, access to immunization, and inadequate response to vaccination are three distinct and equally concerning contributors to poor vaccination coverage in the global population as well as in the vulnerable population. For these and other reasons, personalized vaccine strategies could be considered to improve vaccination coverage and outcome as discussed below.

### 1.2. Reasons to Personalize Vaccine Intervention in Vulnerable Populations

High vaccination coverage is paramount to ensure global health, and it can be achieved by promotion of vaccination and by the design of effective vaccine. However, vulnerable populations consistently generate vaccine-specific immune responses that are considerably weaker than those of the healthy immunocompetent population [1, 2, 12–15]. We previously demonstrated that patients with chronic granulomatous disease (CGD) present a significantly reduced measles-specific antibody levels and antibody-secreting cell number indicating poor ability to maintain long-term memory in these patients [16]. Similarly, we demonstrated that 19% of kidney transplanted patients (TPs) on immunosuppressive therapy experienced loss of vaccine-induced immunity against measles after two doses of live attenuated measles vaccine at 13 months and 6 years of age [17]. Furthermore, we found a positive correlation between the antibody titres and the time elapsed between vaccination and transplant, demonstrating that patients transplanted close to vaccination had lower measles antibody titre than patients vaccinated earlier before transplantation. Reversing this situation is likely to require a broad range of interventions. For example, financial incentives, patient reminders, and patient recall systems can improve vaccination rates and are more readily implemented in high-income country settings [18]. Nonetheless, there is lack of harmonized research data that can provide meaningful evidence on the efficacy and safety of vaccination in this group. Indeed, most vaccine indications in special and vulnerable groups derive from extrapolations, assumptions, or postlicensure studies in healthy populations.

Generating and analysing clinical, laboratory, system biology “OMICs,” and computational data are needed to inform selection of patients at risk for vaccine failure and specifically tailor vaccination approaches in these groups. The number of patients who are immunocompromised is increasing [19], and the suboptimal vaccination coverage in this growing number of people represents a substantial health and economic burden to society, as discussed above. Furthermore, vaccine-preventable infectious diseases have been reported in these groups despite a history of vaccination [19–24]. Such cases are often the first demonstrable sign of inefficacy of the current vaccination strategies in specific populations within a community. This situation has generated major concern in the World Health Organization (WHO) that is promoting strategies (The Guide to Tailoring Immunization Programmes (TIP)) [3] to enhance efficient vaccination in newborns and children, with a plan to extend this action to individuals within other vulnerable populations. Although they are useful, such recommendations are based on expert opinions and extrapolated from data produced in healthy people and not developed based on vaccine immunity data in vulnerable populations.

## 2. Modern System Biology Tools to Characterize Immune Responses to Vaccination

**Figure 1:**
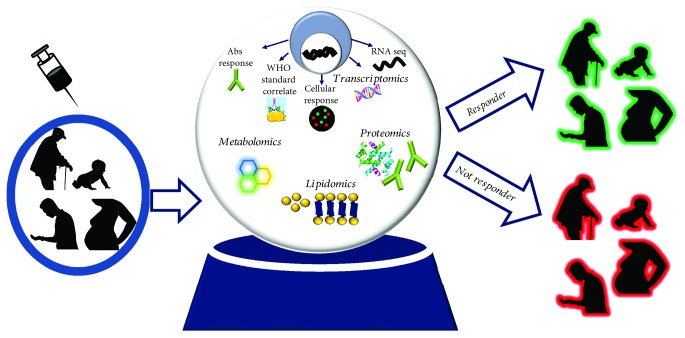
Conventional and system biology “OMICs” technologies [35] currently available to predict vaccine-induced immune response.

Despite the fact that conventional immunological assays, such as ELISA, ELISpot, flow cytometry, and neutralization assays, have supported all previous researches [25–33], the toolkit of the modern immunologists now includes a broad range of “OMICs” technologies [34–37], such as high-throughput sequencing of DNA (DNA-seq), RNA (RNA-seq), transcriptomic assays, microarrays, epigenetics, and high-resolution mass spectrometry proteomics and metabolomics [22, 38–43]. Data produced by these different approaches will enable prediction of patients likely to have a poor outcome from vaccination with respect to safety and/or immunogenicity (Figure 1). The amount of information provided by these experimental approaches represent considerable experimental data analysis challenges [44]; therefore, sophisticated bioinformatic tools are under development for data integration [45].

### 2.1. High-Throughput Sequencing of DNA and RNA (Transcriptomic Assays)

High-Throughput Sequencing of DNA and RNA (Transcriptomic Assays) has helped to identify specific mechanisms that regulate gene expression and associated with differentiation and functionality of different cell lineages including immune cells [39, 46]. For example, Reif et al. identified and validated three SNPs associated with adverse events to smallpox vaccine in healthy vaccinia virus-naïve individuals [47]. The study demonstrated how common genetic variants can be related to a complex clinical phenotype, and prescreening is needed to predict adverse events. Poland and colleagues identified genetic variations in HLA and non-HLA genes associated with non- or hyperimmune phenotypes after measles, mumps, rubella, and smallpox, proposed as “genetic blueprints to guide personalized vaccination regimens” [48, 49]. Other studies have characterized the sequences of heavy and light chains of the antibody following vaccination against pathogens such as influenza and tetanus, with the ultimate aim of engineering responsive antibodies that could be administered to support immunization [50, 51].

Furthermore, DNA sequencing has helped to identify and describe stimulus-induced epigenetic events, paving the way to a new research area: epigenomics. In particular, *DNA methylation* [52, 53] events are associated with (a) differential expression of proinflammatory (IL12p70, IL-1*β*, IL-6, and TNF-*α*) and regulatory (IL-10) cytokines and costimulatory molecules (CD80, CD86, and CD40) in antigen-presenting cells (APCs); (b) regulation of macrophage functional responses and polarization, influencing the innate immune system through macrophage tolerance and training [54]; and (c) modulation of T and B cell differentiation and maturation [54]. Accordingly, recent studies have explored the effect of epigenetic regulation in response to vaccination. For example, individuals showing antimycobacterial activity following BCG vaccination had reduced the presence of methylation events in promoters associated with immune responses in PBMC [55]. In particular, at 3 weeks after vaccination, 540 promoters displayed a more than 5-fold loss of methylation in the responders, whereas only 20 promoters were losing methylation in the nonresponders. Furthermore, at 4 and 8 months, after vaccination, a substantial gain of methylation was observed in the nonresponders. On the contrary, a group of hypomethylated CpGs has been associated with lower humoral immune response to influenza vaccination [56]. Similarly, another study by Marsit et al. [57] demonstrated a small but statistically significant reduction in the methylation of peripheral blood repetitive elements in an HIV-exposed and antiretroviral therapy- (ART-) exposed pediatric cohort when compared with an HIV-exposed and combined ART-unexposed cohort. However, data are still scarce and often contradictory, and efforts are needed to define the power of specific methylation marks in predicting vaccine responses.

The combination of flow-based sorting and microfluidic *transcriptomic assays* (Fluidigm) has enabled dissection of transcriptional signatures of immune cell subsets particularly involved in the memory response upon vaccinations. The low number of cells needed for these assays has made such studies feasible in pediatric cohorts and provides the possibility to investigate gene expression on purified memory subsets rather than in the highly variable pool of PBMCs allowing the analysis of low abundance transcripts. Such methodology increases the specificity of transcriptional characteristics found in peculiar cell subsets which are involved in the immune memory response but are quantitatively rare in the pool of PBMCs [58]. With such strategy, Cotugno et al. have recently investigated the prevaccination gene expression signatures of lymphocyte subsets in groups of HIV-1-infected children differentially responding to trivalent influenza vaccination (TIV). A 25-gene signature in resting memory (RM) B cells (CD27^+^CD21^+^) distinguished vaccine responders from nonresponders (NR). In fact, prevaccination RM B cells of responders demonstrated a higher expression of gene sets involved in B cell adaptive immune responses (*APRIL, BTK, BLIMP1*) and BCR signalling (*MTOR, FYN, CD86*) when compared with NR. We further investigated the variation of gene expression of peripheral T follicular helper (pTfH) cells after *in vitro* stimulation with H1N1 peptides. In line with previous FACS and ELISA results [59], our analysis revealed that the ability to upregulate the gene expression of interleukin-21 (IL-21) within pTfH after *in vitro* stimulation was strongly associated with H1N1-specific B cell responses postvaccination [60]. These results suggest that the targeted transcriptional evaluation of B and T cell subsets at the time of vaccination may identify predictive correlates of vaccine responses in this population. Other advantages of this analytical tool account for containment of costs when compared to RNA-seq (approximately 1/25) and to DNA microarray (approximately 1/10). In addition, the integration and the analysis of targeted multiplexed RT-PCR (e.g., Fluidigm) rather than “big data” deriving from RNA-seq need less sophisticated bioinformatic expertise which may enhance clinical applicability of such analysis.

On the other hand, the selection of specific gene sets for analysis also represents a limitation. Indeed, whole transcriptome or genome analysis may provide more specific and unbiased information on molecular mechanisms underlying vaccine-induced reactogenicity and immunogenicity. In the context of vulnerable populations, such information may provide important input into discovery of specific pathways, inadequately engaged by current vaccines, which may inform future targeted adjuvant strategies. In this context, the interindividual variability in vaccine responses or reaction upon vaccinations has been investigated, and several polymorphisms of genes, including *HLA*, *KIR*, *MICA*, and *BTN* genes, were identified that impact immune responses to immunization against hepatitis B [61–63], influenza [61], and smallpox [64, 65]. Possible mechanisms underlying such correlation presumably refer to the selectivity of specific HLA types to naturally process particular vaccine peptides and present to T and B cells. Such peptides are enriched by specific particles and adjuvants and are now being utilized in a reverse-engineering strategy to develop peptide-based candidates for measles and mumps vaccines [66]. Ovsyannikova et al. recently reported how specific coding polymorphisms in Toll-like receptor (TLR) genes are associated with immunogenicity of measles vaccine [67, 68]. Although these findings represent great steps towards the design of personalized peptides and adjuvants in the immunization schedule for NR, most of these studies have been conducted in healthy individuals (reviewed in [69]). Indeed, such approaches have only rarely investigated vaccine-related immunogenicity and adverse events in vulnerable populations (especially in the elderly) showing how signatures of NR found in healthy individuals are only partially applicable to such populations [70]. However, the few studies conducted on vulnerable populations showed that the genetic signatures associated with lack of vaccine immunogenicity in healthy individuals were not fully powerful when applied to vulnerable populations. Thus, there is an urgent need for more vaccinology studies in these vulnerable populations.

To improve robustness and power of transcriptomic data, gene set enrichment analyses (GSEA) have been developed in order to analyse genes within their functional group or as being part of the same signalling pathway. In line with this approach, increasing numbers of functional annotation tools available online free of charge can identify enriched biological themes—Gene Ontology (http://geneontology.org), DAVID (http://david.abcc.ncifcrf.gov), http://www.pathjam.org, and http://genemania.org—and functionally related gene groups.

In a different approach, the *whole transcriptome* was implemented to describe factors correlated to vaccination immunogenicity in the blood cells of humans few days after yellow fever vaccination [42]. In particular, the authors found enrichment of genes promoting apoptosis including GSTP1, STAT4 inhibitor, IL17D, and ZNF-148 (also known as ZBP-89) (reviewed in [71]). This approach was further explored to define possible correlates of adaptive and innate immunity able to predict immunogenicity of influenza vaccination (live attenuated influenza vaccine and TIV) [37]. Both Nakaya et al. [40] and Tsang et al. [43] found that the calcium/calmodulin-dependent kinase IV (Camk4) gene expression modules could be used as a predictor of low antibody titres upon influenza vaccination. In order to define the vaccine specificity, Li et al. [36] compared five 5different vaccinations and found three different signatures of immune response according to the type of vaccinations used. It is still unclear however, whether gene signatures of vaccine immunity should be investigated in selected lymphocyte subsets or in antigen- (Ag-) specific cells. Technological advances in single-cell analysis allow for deeper interrogation of cellular signatures in cell population with diverse functions, such as Ag-specific cells in memory cell compartments.

Among these, single-cell RNA sequencing (scRNA-seq) [72–74] has provided insights on key processes in immune cell development and differentiation [73, 74], on haematopoietic pathways [75], and on gene regulatory networks that predict immune function [76]. There are multiple scRNA-seq approaches, the most current version being massively parallel RNA single-cell sequencing (MARS-seq), Fluidigm C1 single-cell full length messenger RNA (mRNA) sequencing, switching mechanisms at the 5′ end of RNA template (SMART-seq2), and 10x genomic chromium single-cell DNA sequencing (herein referred to as 10x cell sequencing (reviewed in detail by [74])). Among those, the most promising at the moment is the 10x cell sequencing (described in [77]). This cutting-edge technology performs rapid droplet-basedencapsulation of single cells using a gel bead in emulsion (GEM) approach. Each gel bead is labelled with oligonucleotides that consist of a unique barcode, a 10 bp unique molecular identified and an anchored 30 bp oligo-dT. The high-throughput system is designed to enable analysis of rare cell types in a sufficient heterogeneous biological space avoiding the cell sorting step with reduced waste of the precious clinical sample. Similar to other droplet-based methods, clinical samples must be handled with caution in order to minimize perturbation of existing cellular characteristics [78]. Importantly, this method also enables cellular indexing of transcriptomes and epitopes using DNA-barcoded antibodies by Cellular Indexing of Transcriptomes and Epitopes by sequencing (CITE-seq) of thousands of single cells [79]. Accordingly, CITE-seq could find major applicability in immunology for sequencing of antigen-specific cells by multiplexing specific antigenic protein markers.

### 2.2. Proteomics and Metabolomics

Although high-throughput technologies can provide a valuable “snapshot” of the transcriptional levels of genes inside the cells, the interactions among those genes cannot be fully captured if the above described tools are uniquely used to generate lists of genes or pathways associated to a specific cellular activity. Indeed, it is the functional relationships between genes, proteins, and metabolites that may help us to better understand biological processes involved in cellular responses.

In this optic, the identification of the subset of proteins and peptides involved in the immune response could be pivotal to unravel mechanisms supporting a successful vaccination outcome [80]. Targeted protein analysis assays (e.g., ELISA and WB) only allow for quantification of a certain list of protein candidates limiting the proteomic discoveries. To overcome this hurdle, different high-throughput methods have recently been developed. Mass spectrometry- (MS-) based proteomics is the most widely used approach, and it has been essential to define the major histocompatibility complex (MHC) in the context of T cell profiling [81] as well as the antigenic determinants triggering B cell activity [82–84]. As the MS method is not limited to the use of predefined proteins, it has become the method of choice for protein discoveries across different fields as already been extensively described somewhere else [80]. More recently, Bennike et al. [85] have optimized use of as little as 1 *μ*l of blood plasma for a high-throughput MS approach with bioinformatic analysis employing Spectronaut. This innovative, cost-effective high-throughput technology has, for example, supported the discovery of 16 serum proteins predicting chronic pancreatitis. Indeed, low sample input, high throughput, and robust proteomic depth render this method attractive for large diagnostic studies aiming at the identification of protein biomarkers in different clinical and scientific settings.

Evaluation of metabolomic signatures can be an additional mass spectrometery-based tool to capture perturbation of the immune system after a vaccination and translate such information as potential new biomarkers of vaccine immunogenicity. McClenathan et al. [86] used the nuclear magnetic resonance metabolomic approach to characterize specific metabolites predicting adverse reaction following vaccination. These studies provided a set of metabolites associated with the vaccine outcome that can be used in the clinical practice for identification of vaccine nonresponder individuals. Furthermore, Li et al. applied a multidisciplinary approach to define immunological response to herpes zoster (shingles) by studying transcriptomics, metabolomics, plasma cytokines, and cell phonotypes in blood samples.

### 2.3. Data Integration

OMIC approaches have changed perspectives and dimension of data to be handled and interpreted. Indeed, most of these sophisticated approaches often require big sample volume, which may hurdle the large-scale applicability of the methods. The Human Immunology Project Consortium (HIPC, https://www.immuneprofiling.org [87]) program has developed novel analytic tools to integrate the information derived from OMICs, *in vitro* assays, and functional assays to define vaccine responsiveness.

The overwhelming amount of data represents both a precious source and a hurdle towards the design of rule-driven precision medicine [34]. Indeed, there is the need for more complex algorithms capable of integrating data from different system biology approaches that will consequentially be implemented, tested, and validated in order to generate a clinical tool that can support the personalization of vaccination strategy. Accordingly, novel research approaches in the last two decades have led to partnerships of basic scientists, bioinformaticians, and physicians to appropriately interpret data. With this aim, specific tools have been developed to enable gene set enrichment analyses (GSEA) in order to improve robustness, power, and readability of transcriptomic data, as mentioned above. Furthermore, there are various modelling frameworks that can be applied which range from simple linear regression models to advanced and computationally expensive feature selection methods for identifying predictive signatures (reviewed in [88]). For example, network modelling provides a powerful way to uncover the organizing principles and regulatory elements of cellular networks and how these networks modulate immunological responses to vaccination (reviewed in [88]). Additional tools such as Network Analyst and DIABLO (Data Integration Analysis for Biomarker discovery using Latent variable approaches for ‘Omics studies) have been employed to understand multidimensional data across multiple assay platforms [45].

## 3. Ebola and Influenza Vaccines: Two Successful OMIC Examples of Applied System Vaccinology

### 3.1. Ebola

Rechtien et al. applied a system vaccinology approach to unravel if the early immune response towards Ebola vaccine rVSV-Zaire Ebola virus (ZEBOV) predicts the generation of anti-Ebola virus (EBOV) glycoprotein- (GP-) specific antibody responses [89]. The study employed blood samples from days 0, 1, 3, 7, and 14 postvaccination to investigate changes in cytokine levels, innate immune cell subsets, and gene expression. Integrative statistical analyses with cross-validation identified a signature of 5 early innate markers correlating with antibody titres on day 28. Among those, interferon-*γ*-inducible protein 10 (IP-10) on day 3 and MFI of CXCR6 on NK cells on day 1 were independent correlates. Consistently, they found an early gene expression signature linked to IP-10. This comprehensive characterization of early innate immune responses to the rVSV-ZEBOV vaccine in humans revealed immune signatures linked to IP-10. These results suggested correlates of vaccine-induced antibody induction and provide a rationale to explore strategies for augmenting the effectiveness of vaccines through manipulation of IP-10.

### 3.2. Influenza

In line with our data on influenza previously discussed [22, 60, 90], Franco and colleagues studied a homogenous population of 199 healthy male volunteers with trivalent influenza vaccines [38]. They performed an integrative genomic analysis of the human immune response to influenza vaccination exploring association of genotype to gene expression, gene expression to antibody titre, and genotype to antibody titre. They identified 20 genes associated with a transcriptional response to vaccination, significant genotype effects on gene expression, and correlation between the transcriptional and antibody responses. The following loci were found to have the strongest evidence of genetic variation influencing the immune response to the vaccine: *TAP2*, *SNX29*, *FGD2*, *NAPSA*, *NAPSB*, *GM2A*, *C1orf85*, *JUP*, *FBLN5*, *CHST13*, *DIP2A*, *PAM*, *D4S234E*, *C3AR1*, *HERC2*, *LST1*, *LRRC37A4*, *OAS1*, *RPL14*, and *DYNLT1*. The results showed that variation at the level of genes involved in membrane trafficking and antigen processing significantly influenced the human response to influenza vaccination. Overall, this study identified crucial genes in the humoral response to vaccination suggesting such marks as logical biomarkers predicting the vaccination outcome. Such examples show how OMICs can be used to predict vaccination outcome in order to identify nonresponders.

## 4. Rationalising the Development of Adjuvants as Possible Strategy to Personalize Vaccines

In order to improve the efficacy of the vaccine, adjuvants can be added to antigens in order to stimulate in a selective way the different routes of innate and adaptive immunity [34]. The use of optimized adjuvanted formulations may overcome host characteristics that limit vaccine response and possibly favour personalized vaccine interventions. Adjuvants can be crucial to enhance immune response in low-responder individuals. Reference [91] explored the potentiality of TLR8 agonist as adjuvant for BCG and pneumococcal vaccination in newborns. TLR8 agonist-encapsulating polymersome triggered dendritic cell (DC) responses enhancing vaccine immunogenicity, thus suggesting TLR8 potential for early-life immunization against intracellular pathogens. Adjuvants may be delivered as components of microorganisms. For example, *Neisseria meningitidis* lipopolysaccharide (LPS) is a good example. Mehta et al. demonstrated that LPS exhibited differential adjuvant properties when formulated as native outer membrane vesicles (nOMVs). nOMVs enhanced immunogenicity suggesting that they may be an effective adjuvantation approach for future meningococcal protein vaccines. [92].

By combining OMIC technologies, data on vaccine immunity in groups with special vaccination needs, and adjuvant screening and development, we can increase our knowledge on mechanisms of vaccine hyporesponsiveness and how to overcome it. In the near future, these efforts will enable a new generation of adjuvants designed to stimulate, in a selective way, the different routes of innate and adaptive immunity.

## 5. Future Perspectives: From Vulnerable One-Fits-All Vaccines towards Invulnerable One-Fits-One Vaccines

**Figure 2:**
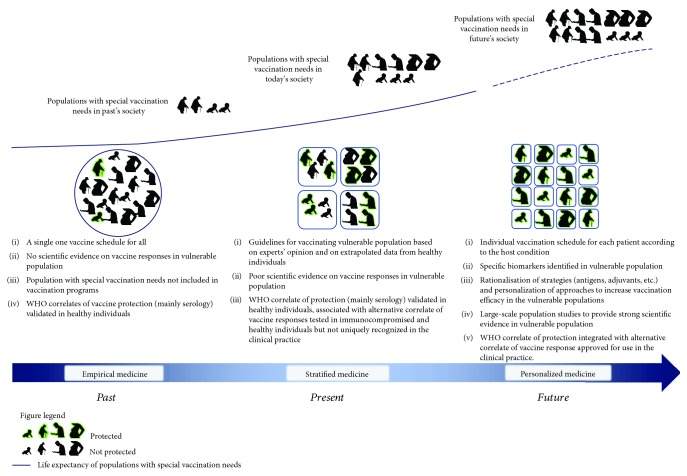
The figure shows changes in populations' composition with special vaccination needs over time. Traditionally, only elderly people and infants were considered vulnerable. During this time period, little scientific evidence regarding vaccine responses of different populations was available, and a single vaccination schedule was proposed for all (empirical medicine). Currently, an increasing number of people with special vaccination needs, such as immunocompromised patients and pregnant women, are considered in specific vaccine programs based on expert opinion and on extrapolated data from healthy individuals (stratified medicine). In the near future, the increased number and life expectancy of groups with special vaccination needs will lead to large-scale population studies. This approach will provide robust scientific evidence and new correlates of protection and safety. As a result, rationalisation of vaccine strategies (antigens, adjuvants, etc.) and personalization of approaches will increase vaccination efficacy and safety in these populations within a framework of personalized medicine.

Vaccines have greatly improved life expectancy by containing and in some cases eradicating diseases causing pathogens. Preventing vaccine disease has great impact not only on global health but also on the economy of the society 5by reducing hospitalization costs. Originally, one single vaccine was developed to target the global population accounting for limited cases of vaccine failure, even though data on vaccine failure was scarce especially in vulnerable populations (Figure 2). However, this approach is becoming less successful with the expansion of a population of immunocompromised individuals that fail to respond to standard vaccination schedules and compositions. Therefore, vaccinology is in part focused on tailoring specific interventions for these vulnerable individuals in the near future.

At the moment, there are some indications on how to optimize vaccine strategies in vulnerable individuals. However, interventions are still decided upon evidence deriving from study of healthy individuals or upon expert's opinion. To improve on this current approach, current efforts aim to better characterize the vulnerable population, which can be integrated to generate predictive bioinformatic models for precise early identification of nonresponders. System biology studies are already revealing genetic and molecular “signatures” of protective immune response in healthy population [93]. In the near future, we trust that it will be possible to narrow such signatures to highly predictive assays of efficacy/effectiveness and identify precise correlates of protection in vulnerable groups (Figure 2).
